# Gene targeting in adult rhesus macaque fibroblasts

**DOI:** 10.1186/1472-6750-8-31

**Published:** 2008-03-26

**Authors:** Daniel T Meehan, Mary Ann Zink, Melissa Mahlen, Marilu Nelson, Warren G Sanger, Shoukhrat M Mitalipov, Don P Wolf, Michel M Ouellette, Robert B Norgren

**Affiliations:** 1 Genetics, Cell Biology and Anatomy, University of Nebraska Medical Center, 985805 Nebraska Medical Center, Omaha, NE 68198-5805, USA; 2 Department of Pediatrics and Human Genetics Laboratory, Munroe Meyer Institute for Genetics and Rehabilitation, University of Nebraska Medical Center, Omaha 68198-5440, USA; 3 Oregon National Primate Research Center, Oregon Stem Cell Center, Oregon Health & Science University, 505 NW 185th Ave, Beaverton, OR 97006, USA; 4 Eppley Institute for Research in Cancer, University of Nebraska Medical Center, Omaha, NE 68198, USA

## Abstract

**Background:**

Gene targeting in nonhuman primates has the potential to produce critical animal models for translational studies related to human diseases. Successful gene targeting in fibroblasts followed by somatic cell nuclear transfer (SCNT) has been achieved in several species of large mammals but not yet in primates. Our goal was to establish the protocols necessary to achieve gene targeting in primary culture of adult rhesus macaque fibroblasts as a first step in creating nonhuman primate models of genetic disease using nuclear transfer technology.

**Results:**

A primary culture of adult male fibroblasts was transfected with hTERT to overcome senescence and allow long term *in vitro *manipulations. Successful gene targeting of the HPRT locus in rhesus macaques was achieved by electroporating S-phase synchronized cells with a construct containing a SV40 enhancer.

**Conclusion:**

The cell lines reported here could be used for the production of null mutant rhesus macaque models of human genetic disease using SCNT technology. In addition, given the close evolutionary relationship and biological similarity between rhesus macaques and humans, the protocols described here may prove useful in the genetic engineering of human somatic cells.

## Background

Rhesus macaque models of human genetic disease would be very useful for translational research. Although mouse "knockouts" have provided much basic information regarding the effects of gene disruption, for some genes, null mutations in the mouse ortholog of a human disease gene does not result in a phenotype that completely matches the human disease. Thus, there are either no or limited animal models for many human genetic diseases [1]. Furthermore, non-human primates are often preferred for translational research due to close similarities to humans in behavior, anatomy, physiology, and genetics [2-6].

Currently, the vast majority of genetic models of human disease in mammals have utilized the mouse as an experimental animal [7]. This is due to the relative ease with which genetically modified animals, including loss-of-function and knock-in mutations, can be produced by transfecting ES cells and creating chimeric animals with germline changes [7]. Unfortunately, although ES cells have been derived from primates including rhesus macaques, they have not been shown to contribute to chimeras.

Null mutant sheep, goats, pigs and cattle have been produced using an alternative approach: gene targeting in somatic cells followed by nuclear transfer to enucleated oocytes (SCNT; reproductive cloning) [8-18]. Gene targeting in human fibroblasts has also been achieved [19-25]. Because many different experimental protocols have been applied to achieve gene targeting in mammalian fibroblasts, we wished to determine which protocols would support efficient gene targeting in rhesus macaque fibroblasts as a first step in creating null mutant rhesus macaques using SCNT.

The HPRT gene was the first gene disrupted in the mouse [26]. There were several reasons for this, including the fact that mutations in the human ortholog results in a severe neurodevelopmental disorder known as Lesch-Nyhans disease, and the fact that mouse HPRT null ES cells could be selected for by adding compounds that would kill any cells expressing the HPRT protein [27-29]. Moreover, HPRT is located on the X chromosome; therefore disruption of only one copy of the gene in male XY cells is sufficient to generate a phenotype. Unfortunately, although mice with HPRT null mutations did exhibit a 50% loss of dopamine in the striatum [30], they did not exhibit the same spontaneous behavioral abnormalities seen in humans with Lesch-Nyhans disease [31]. We chose to target the HPRT gene in male rhesus macaque cells for three reasons: we could select for null mutant cells in culture; it would only be necessary to disrupt one allele to observe a phenotype; and because an animal model with the same behavioral deficits seen in Lesch-Nyhan's disease does not currently exist.

Several different techniques, and combinations of techniques, were evaluated before we determined a set of protocols that resulted in efficient production of gene targeted fibroblasts in rhesus macaques. In this report, we describe what did and did not work for us. One of the important keys to our success was the use of cell cycle synchronization prior to transfection and the inclusion of a SV40 enhancer in the targeting construct [32].

## Results

### hTERT transfection of fibroblasts

**Figure 1:**
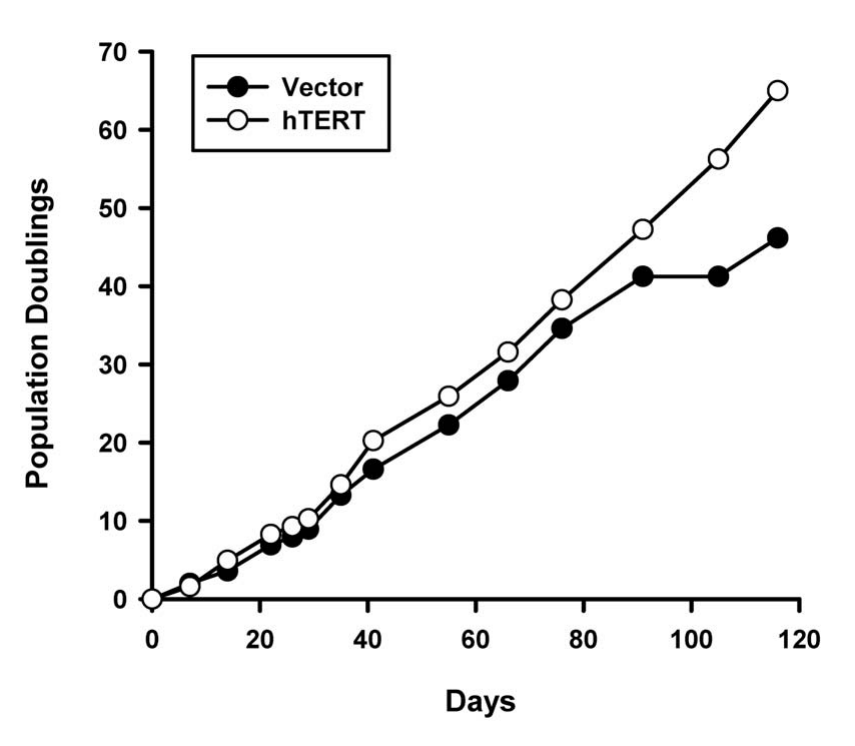
**Extension of lifespan by exogenous telomerase**. Early passage rhesus macaque fibroblasts were transfected with vector pCi-neo carrying no insert (Vector) or the hTERT cDNA (hTERT). Transfected cells were maintained in log phase growth and counted at the time points shown, with the age of each culture expressed in population doublings.

In preliminary studies with rhesus fibroblasts, we had difficulty maintaining these cells in culture long enough to support gene targeting procedures due to senescence. hTERT transfection has been shown to prevent senescence in rhesus fibroblasts [33,34]. To extend the time that fibroblasts could be cultured, we electroporated adult male rhesus macaque fibroblasts with a construct containing a human telomerase reverse transcriptase (hTERT) expression cassette (pCI-neo-hTERT, courtesy of Dr. Robert Weinberg). After G418 selection, cells were passaged continuously until after the empty vector control fibroblasts reached senescence. Control fibroblasts entered senescence after 46 population doublings (Figure 1) whereas the hTERT-transfected cells showed no change in growth rate, no evidence of senescence, and were still dividing after more than 24 weeks (Figure 1).

**Figure 2:**
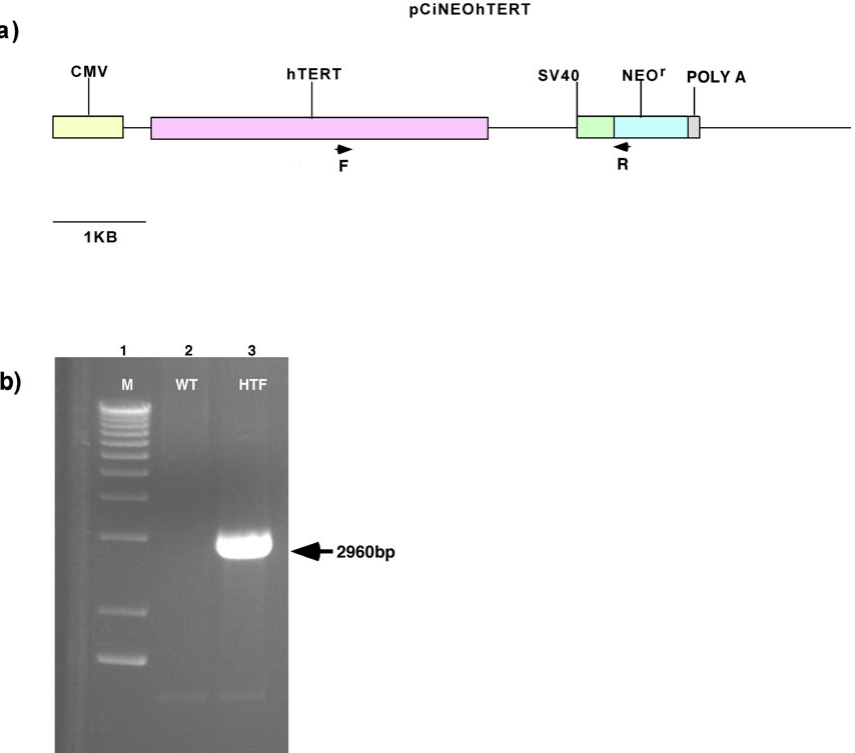
**hTERT genomic PCR assay**. a) pCI-neo-hTERT construct. Primers (arrows) amplify a 2960 bp product that includes the 3' end of the hTERT cassette and the 5' end of the NEO^r ^cassette. b) M = 1 kb Marker, WT = wild type rhesus fibroblasts, HTF = TERT transfected fibroblasts (Experiment #8, HPRT null clone).

**Figure 3:**
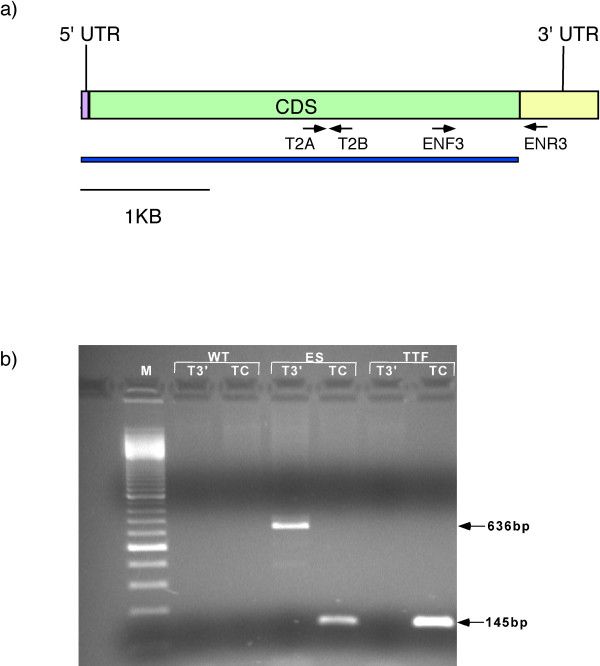
**RT-PCR for TERT expression in rhesus fibroblast cells**. a) CDS = coding region, T2A = hTERT-T2A, T2B = hTERT-T2B, ENF3 = MK TERT ENF3, ENR3 = MK TERT ENR3, blue line = portions of TERT included in pCI-neo-hTERT construct. b) M = 100 bp marker; WT = wild type rhesus fibroblasts; ES = rhesus embryonic stem cells; TTF = TERT transfected fibroblasts (experiment #8, HPRT null clone) T3' = Amplicons of 3' UTR TERT found in endogenous TERT mRNA but not present in construct hTERT mRNA; TC = Amplicons of coding TERT found in both endogenous and construct hTERT mRNA.

To verify that the hTERT-transfected fibroblasts carried integrated copies of the transfected hTERT gene, genomic DNA was extracted and PCR analysis was performed to screen for the presence of the construct (Figure 2A). The PCR assay demonstrated that hTERT was likely stably integrated in the rhesus fibroblast genomic DNA (Figure 2B). To determine if hTERT was expressed in the rhesus fibroblasts, RNA was extracted from NEO^r ^cells and reverse transcribed to cDNA. Two sets of primers were used. One set of primers amplified a 145 bp fragment from the coding region. The other set of primers amplified a 636 bp fragment which included a portion of the TERT 3' UTR not included in the construct. This made it possible to use a reverse primer that would anneal to wild type TERT transcript but not transcript from the construct (Figure 3).

We were able to further test whether the TERT expressed in the transfected rhesus macaque fibroblasts originated from the introduced construct or the endogenous gene by sequencing the RT-PCR product. This was possible because pCI-neo-hTERT contains the human TERT cds, which differs from rhesus macaque TERT in 3 positions of the 145 bp coding amplicon described above. Sequencing of this amplicon indicated that the TERT expressed in the transfected rhesus macaque fibroblasts was derived from pCI-neo-hTERT, not the endogenous TERT gene.

A second population of hTERT-transfected fibroblasts was also made, which we maintained under selection for 20 weeks. In the 8 gene targeting experiments represented in Table 1, either the first (Exps. 1–7) or the second (Exp. 8) population of hTERT-transfected fibroblasts were used. In general, pCI-neo-hTERT transfected fibroblasts generated a greater average number of PURO^r ^colonies than wild type fibroblasts after being electroporated with the targeting construct (84 colonies with wild type cells vs. a mean of 146 colonies with TERT-transfected cells {experiments 2–5}). After S-phase synchronization/electroporation (see below), pCI-neo-hTERT transfected fibroblasts returned to a proliferative state sooner than wild type fibroblasts. A total of 8 targeting experiments were performed with the hTERT-transfected fibroblasts, which yielded 21 colonies displaying the HPRT null phenotype (Table 1). An attempt to target wild type fibroblasts was unsuccessful (data not shown).

**Table 1:** Results of targeting experiments with the pHPRT PURO SV40 vector in S-phase cell synchronized hTERT transfected fibroblasts.

**Exp**	**# of cells**	**PURO**^r^**+**	**8AG**^r^**+**	**6TG**^r^**+**	**PCR +**	**Karyotype**
1	10^7 ^cells	NC*	1	1	1	Abnormal
2	10^7 ^cells	160	2	2	2	ND**
3	10^7 ^cells	170	7	7	6	ND**
4	10^7 ^cells	132	6	6	6	ND**
5	10^7 ^cells	122	3	3	2	ND**
6	10^7 ^cells	221	1	1	1	Normal
7	10^7 ^cells	152	0	0	0	ND**
8	10^7 ^cells	410	1	1	1	Normal

*NC = not counted, **ND = not done

### Transfection strategies

To compare transfection methods, rhesus macaque fetal fibroblast cells were transfected with linearized pCMV-Sport-β-gal (Gibco Life Technologies, Grand Island, NY) using three commercially available cationic lipids: Lipofectamine (Life Technologies, Inc., Gaithersburg, MD); SuperFect (Qiagen, Hilden, Germany); and FuGENE6 (Roche Diagnostics Corporation, Indianapolis, IN). The results of these chemotransfection methods were compared with electroporation. With Lipofectamine, only one β-Gal positive cell out of 300,000 was seen 7 days after transfection. With SuperFect, no β-Gal expressing cells were observed 7 days after transfection. FuGENE6 transfection resulted in .02% β-Gal positive cells 7 days after transfection. FuGENE6 was also less toxic to cells. In contrast, electroporation resulted in 1 – 5% of the transfected cells expressing β-gal after 7 days. These results, and a report by Zwaka and Thomson [35] indicating that the chemical methods of transfection were not effective for gene targeting in human ES cells, prompted us to focus on electroporation for our gene targeting experiments.

To achieve gene targeting in adult rhesus macaque fibroblasts, several different strategies for construct design were implemented. Two promoterless constructs were engineered, one with a neomycin resistance (NEO^r^) selection cassette and the other with a puromycin resistance (PURO^r^) selection cassette. Both contained an internal ribosomal entry site (IRES). In targeting experiments utilizing these constructs, no apparent NEO^r ^or PURO^r ^colonies were observed (Table 2). Electroporation of fibroblasts with a targeting construct containing a phosphoglycerate kinase (PGK) promoter and the neomycin cassette resulted in numerous G418 colonies. However, none were resistant to 6TG, indicating that the HPRT gene had not been targeted (Table 2).

**Table 2:** Results of conventional HPRT targeting strategies in hTERT transfected fibroblasts

**Vector**	**Promoter**	**IRES**	**Antibiotic**	**Colonies^r^/5 × 10^6 ^cells**	**Targeting events**
pMK HPRT INEO	None	Yes	NEO	0	0
pMK HPRT IPURO	None	Yes	PURO	0	0
pHPRT+/-	PGK	No	NEO	282	0

Our failure to achieve gene targeting in rhesus macaque fibroblasts using conventional approaches prompted us to consider alternative methods. Mir and Piedrahita [32] discussed the difficulties in obtaining gene targeting in somatic cells and reported that two techniques, thymidine block of cell cycling and the inclusion of a SV40 enhancer sequence in a vector, dramatically improved gene targeting in somatic cells. This motivated us to test these techniques with our pCI-neo-hTERT transfected rhesus fibroblasts.

### S-phase Synchronization

**Figure 4:**
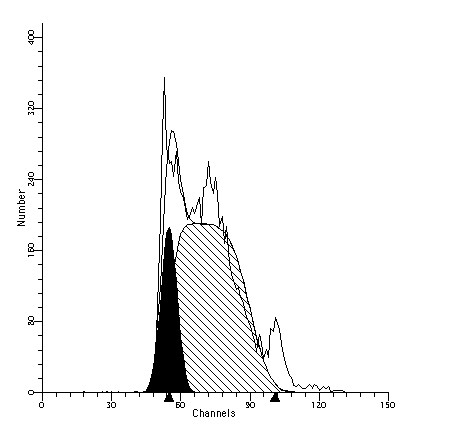
**Cell cycle analysis**. DNA histogram of rhesus fibroblasts stained with Vindelov's reagent after 24 hour treatment with thymdine (Cell Analysis Facility, UNMC). The percentage of cells in S phase as obtained by the computer fit model of Modfit LT. G0/G1: 19.56%, G2/M: 0%; S: 80.44%

We performed a cell cycle analysis of adult rhesus macaque fibroblasts. Cells were treated with thymidine for 12, 18, 24, 30 and 36 hours and harvested for cell cycle analysis. Thymidine treatment for 24 hours resulted in the highest number of cells in S-phase (80%) (Table 3; Figure 4).

**Table 3:** Percentage of fibroblasts in the S-Phase of the cell cycle by a 2 mM thymidine treatment for times ranging from 12 to 36 hours.

*Hours*	*12*	*18*	*24*	*30*	*36*
**% Cells S-Phase**	21%	27%	80%	45%	14%

To test the hypothesis that S-phase arrested rhesus macaque fibroblasts would reduce random integration of the targeting construct, we synchronized cells with thymidine treatment and then transfected them with a targeting construct containing the neomycin resistance gene driven by the PGK promoter. This hypothesis was confirmed as we observed a 28 fold reduction in the rate of random integration of the targeting construct after S-phase synchronization as compared to non-synchronized cells. However, no targeted events occurred in either experiment (Table 4).

**Table 4:** Reduction of random integration by S-phase synchronization

**Targeting Vector**	**S-phase synchronization**	**# of cells**	**G418**^r^**colonies**
pHPRT+/-	No	10^7 ^cells	564
pHPRT+/-	Yes	10^7 ^cells	20

The targeting vector used in this experiment did not contain the SV40 enhancer.

### Targeting construct with SV 40 enhancer element

**Figure 5:**
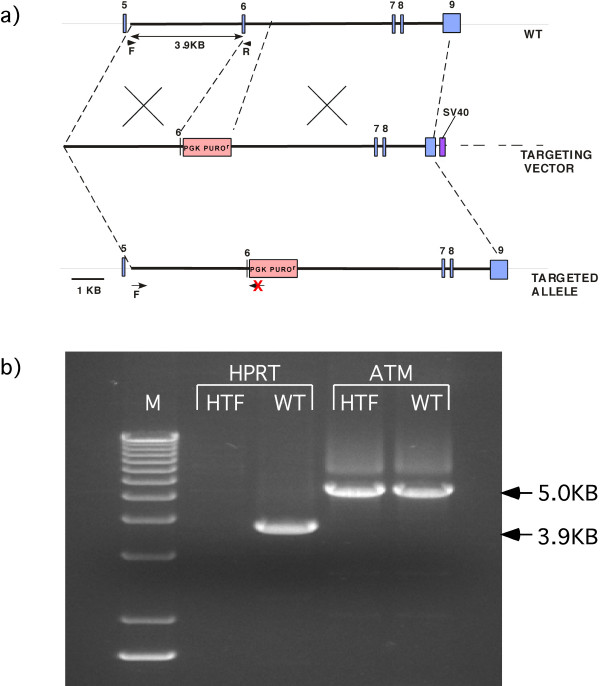
**PCR screening strategy**. a) A 3.9 kb PCR band occurs in the non-targeted wild type rhesus macaque fibroblast DNA. The reverse screening primer anneals to the deleted part of exon 6. If a targeting event has occurred, there will be no 3.9 kb PCR band since that part if exon 6 has been deleted. b) M = 1 kb Marker, HTF = HPRT targeted fibroblasts, WT = wild type rhesus fibroblasts, ATM = ataxia telangiectasia mutated (positive control).

An HPRT targeting construct with an SV40 nuclear localizing DNA was created (Figure 5A). The inclusion of the SV40 nuclear localizing DNA was previously reported to increase gene targeting in bovine fibroblasts, especially when used in conjunction with S-phase synchronization [32]. This construct contained a total of 10.3 Kb of homology to the HPRT locus, 3.9 Kb at the short arm and 6.4 Kb at the long arm. The HPRT sequence was isogenic with the fibroblasts used for targeting. The selection cassette included the puromycin resistance gene driven by the PGK promoter.

### Transfection and selection

We utilized the Mir and Piedrahita [32] recommendation of 4 pulses of electroporation at 450 V, which represented a significant change from our normal electroporation parameters. After electroporation, cells that were thymidine blocked did not divide for four days; therefore, puromycin selection was not initiated until 4 days after electroporation. The specific sequence and timing of selection was crucial in identifying HPRT-null (targeted) fibroblasts. Critical parameters that were identified by Dr. Piedrahita (personal communication) included selection on 96 well plates at 5,000 cells per well (low cell density) and the use of puromycin selection. Selection for HPRT null mutant cells was more effective when 8AG selection preceded 6TG selection.

### Generation of HPRT null mutant rhesus macaque fibroblasts

The combination of cell synchronization and the electroporation of pCI-neo-hTERT transfected fibroblasts with a targeting construct containing the SV40 enhancer resulted in targeting of the HPRT locus in rhesus macaque fibroblasts (Table 1). A total of 8 targeting experiments were performed; 21 colonies had the HPRT null phenotype.

In order to screen for targeted clones, primers were designed that produced an amplicon if the gene was not targeted and did not produce an amplicon if the gene was targeted (Figure 5). Nineteen of these colonies were verified by PCR to be targeted (Table 1). Of the remaining 2 colonies, one was further analyzed and found to contain an insertion of exons 7 and 8 from the targeting construct into the HPRT locus, without replacement, causing a frameshift that resulted in a stop codon at exon 7.

PCR analysis of genomic DNA demonstrated the persistence of the pCI-neo-hTERT in HPRT targeted clones generated in experiments 1 and 8 (Table 1). RT-PCR demonstrated TERT expression in the HPRT null clones from experiments 1 and 8 (Figure 3B), but not experiment 6.

**Figure 6:**
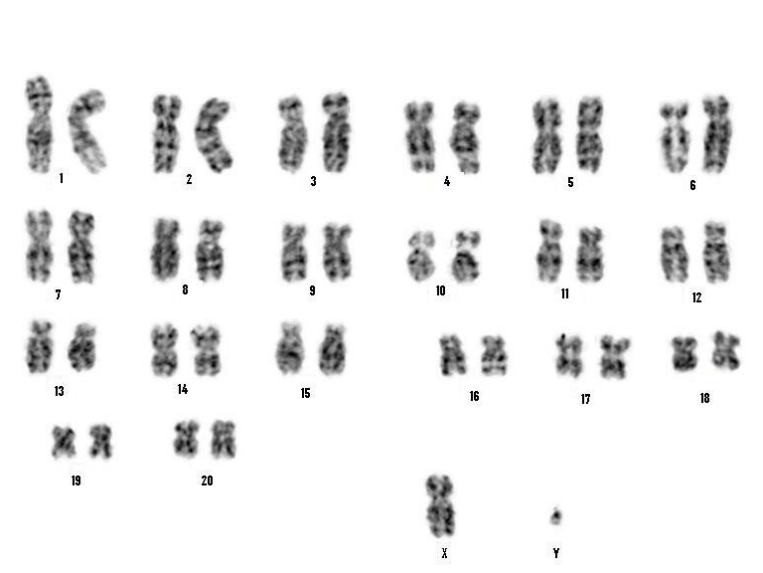
**Karyotype analysis of HPRT targeted cells**. G-banded karyotype from the HPRT null clone (from experiment 8) showing a normal male rhesus macaque chromosome complement.

Cytogenetic analysis indicated that the HPRT null clone from experiment 1 had an abnormal karyotype involving a balanced translocation of chromosome 19 (data not shown). However, targeted clones from experiments 6 and 8 (Figure 6) were karyotypically normal.

An attempt to target wild type fibroblasts was unsuccessful.

## Discussion

When deciding how to disrupt a gene in a mammalian somatic cell, there are a number of choices the investigator must make. Which method of transfection should be used? Which type of vector design and culture conditions should be chosen to enrich for targeted cells? An additional issue which must be addressed for rhesus macaques is: how to obtain a sufficient supply of fibroblasts to successfully target a gene given that many of the transfected cells will become senescent prior to completion of selection procedures. We found that identifying the right combination of approaches to successfully disrupt the HPRT gene in fibroblasts in rhesus macaques to be quite challenging.

There are three main approaches to transfecting cells for gene targeting: chemical, electroporation and viral. The success rate of these different approaches has varied greatly depending on the technique and in which lab the targeting occurred. For example, efficient gene targeting has been reported with chemical transfection methods in fibroblasts from sheep [14] and pigs [36,37]. Electroporation has been used to introduce targeting constructs into fibroblasts from humans [20], sheep [11], pigs [9,10,38,39] and cows [17,32]. Adeno-associated vectors have been used for gene targeting in human [21] and cow [40] somatic cells.

Although we have not systematically tested all possible methods of gene targeting in rhesus macaques, we did examine a number of different methods. We initially attempted gene targeting in rhesus macaque fibroblasts using chemical methods. These attempts were unsuccessful. Others have also reported difficulties with this approach for gene targeting [35]. We were able to achieve successful gene targeting with electroporation, but only after utilizing a SV40 enhancer in the targeting construct and synchronizing the cell cycle of the fibroblasts.

It is difficult to explain why gene targeting approaches that are reported to be very efficient in one lab are not successful in another. When we applied different methods of gene targeting to rhesus fibroblasts, we followed the recommended protocols for other species. In our hands, only one approach was successful for rhesus macaque fibroblasts [32]. It is possible that modifications of the other protocols would have resulted in success in rhesus macaques. Although we did not attempt the use of viral vectors for gene targeting in rhesus macaque fibroblasts, their successful use in human and cow somatic cells suggests that this would be a possible method for performing gene targeting in rhesus macaque fibroblasts [21,40].

Fetal fibroblasts are used for gene-targeting in studies for most mammals. For rhesus macaques, obtaining a sufficient supply of such cells for experimentation is difficult. Further, in our preliminary experiments with fetal fibroblasts, we found that many of these cells were becoming senescent towards the end of the selection procedures. Extending the lifespan of adult fibroblasts by transfecting them with TERT, allowed us to obtain a sufficient supply of cells to test various gene targeting methods and avoid senescence. It should be noted that in one of our targeted clones (experiment 6, Table 1), hTERT was no longer expressed. It is possible that the construct was never integrated in this clone or that it was silenced at some point during gene targeting selection.

Long term culture of somatic cells necessary for gene targeting can result in chromosomal abnormalities. In our study, a chromosomal translocation was observed in one of the targeted clones (data not shown). However, we were able to produce targeted clones that did not exhibit any detectable karyotypic abnormalities (Figure 6). It is possible that the expression of hTERT in the pCI-neo-hTERT transfected fibroblasts protected them from undergoing crisis and transformation.

The current studies provide a workable protocol for producing genetically modified non human primate (NHP) fibroblasts. However, before these cells can be used for SCNT it will be necessary to remove the hTERT construct. hTERT expressing sheep fibroblasts have been shown to result in blastocysts at normal rates after SCNT, but fetuses did not survive past 40 days of development suggesting that the hTERT may have negatively impacted development at this stage [41]. In addition, constitutive expression of hTERT might complicate phenotype analysis. Dysregulation of TERT is thought to be a contributing factor in cancer development [42]. Thus, one approach to creating gene targeted NHPs will be to introduce TERT into fibroblasts, perform gene targeting, remove the TERT vector, confirm euploidy and then perform SCNT.

## Conclusion

Although many protocols have been published for gene targeting in mammalian somatic cells, most were ineffective in our hands when we attempted to disrupt the HPRT gene in rhesus macaque fibroblasts. The critical parameters for successful gene targeting in primary culture of rhesus macaque fibroblasts included hTERT transfection, cell synchronization in S-phase and inclusion of a SV40 enhancer in the targeting construct. The cell lines reported here, with the removal of the TERT vector, could be used for the production of null mutant rhesus macaque models of human genetic disease using SCNT technology. In addition, given the close evolutionary relationship and biological similarity between rhesus macaques and humans, the protocols described here may prove useful in the genetic engineering of human somatic cells.

## Methods

### Generation of hTERT transfected fibroblasts

Early passage of primary culture fibroblasts derived from an ear biopsy of a 16 year old male rhesus macaque (animal #20109, Oregon National Primate Research Center) were grown to confluency on a 10 cm tissue culture dish (Becton Dickenson, Franklin Lakes, NJ). Cells were trypsinized and electroporated at 0.260 kV, 1 × 10^3 ^μF in 500 μl of basal DMEM (Gibco, Carlsbad, CA) at 4°C with 10 μg of linearized pCI-neo-hTERT (courtesy of Dr. Robert Weinberg). Transfected cultures were selected with 400 μg/ml Geneticin (G418 – Gibco, Carlsbad, CA) and subsequently passed at confluency over the course of 24 weeks. Low passage NEO^r ^cells were frozen for future use.

Clonal hTERT expressing cell lines were generated by electroporating wild type fibroblasts with four pulses of 0.450 volts, 50 μF and then plating at 5,000 cells/well on 96-well plates with Geneticin containing media.

### PCR screening of rhesus fibroblasts for presence of the hTERT construct

Genomic DNA was extracted from pCI-neo-hTERT transfected fibroblasts that had been passaged 13 times. PCR was performed with 200 ng of genomic DNA (purified with the PureGene Genomic Purification Kit, Gentra Systems, Minneapolis, MN according to manufacturer's instructions) and primers TERT/NEO F1(5'-AACGTTCCGCAGAGAAAAGA-3') and TERT/NEO R1(5'-TGTCTGTTGTGCCCAGTCAT-3'). PCR conditions were as follows: Initial denaturing step, 95°C for 2 minutes; 34 cycles of 95°C for 30 seconds, 56°C for 30 seconds and 72° for 3 minutes; 72°C for 7 minutes. The expected size of the PCR product was 2.9 kb.

### RT-PCR analysis of expressed TERT

Analysis of TERT expression was performed with 2 μg of rhesus RNA isolated from embryonic stem cells (ORMES-6, Oregon National Primate Research Center), wild type fibroblasts, and pCI-neo-hTERT transfected fibroblasts. RNA was extracted using Trizol (invitrogen, Carlsbad, CA) as recommended by the manufacturer. DNase1 (Invitrogen, Carlsbad, CA) was used to remove any contaminating DNA. RNA was then reverse transcribed according to the manufacturer's protocol with Superscript III (Invitrogen, Carlsbad, CA). PCR was performed on the cDNA using FastStart High Fidelity Polymerase (Roche Applied Science, Indianapolis, IN). Primers MK TERT ENF3 (5'-TCCTGCTCAAGCTGACTCAA-3') and MK TERT ENR3 (5'-AAGGTCAGGGTGATGAGTGG-3') amplified a 636 bp fragment expressed by the endogenous TERT gene but not pCI-neo-hTERT. Primers hTERT-T2A (5'CGGAAGAGTGTCTGGAGCAA-3') and hTERT-T2B (5'-GGATGAAGCGGAGTCTGGA-3') amplified a 145 bp TERT coding sequence fragment expected to be expressed by both the endogenous TERT gene and pCI-neo-hTERT. PCR conditions were as follows: Initial denaturing step, 95°C, 2 minutes; 34 cycles of 95°C for 30 seconds, 53°C for 30 seconds and 72°C, 1 minute; 72°C for 7 minutes.

### Construction of a rhesus macaque HPRT targeting vector

The targeting vector was designed to delete 32 base pairs of the rhesus HPRT exon 6 and replace it with a puromycin resistance (PURO^r^) gene driven by the phosphoglycerate kinase (PGK) promoter. This selection cassette was PCR amplified from pKO Select PURO (Stratagene, LaJolla, CA). Both the 5' (short arm) and the 3' (long arm) of the homologous regions of the construct were PCR amplified from genomic DNA obtained from an adult male rhesus macaque (animal # 20109, Oregon National Primate Research Center). Total length of homology was 10.3 Kb. An SV40 sequence was inserted 3' to the 3' arm to increase nuclear import of the construct. pKO Scrambler V916 (Stratagene, La Jolla, Ca) was used as the backbone for the targeting construct.

The 5' end of the construct, containing a 3.9 Kb fragment that spanned the 3' end of intron 5 to the 5' end of exon 6, was amplified with the primer pair HPRT ShortArm, forward 5'-GGAAGATCTTAATGGTGGACTTGTGTTCTAA-3' and HPRT ShortArm, reverse 5'-GGAAGATCTCCTGACCAAGGAAAGCAAAG-3'. Due to a high GC content in the amplified region, the Advantage GC Genomic Polymerase Mix (BD Biosciences Clontech, Mountain View, CA) was used for this PCR. PCR conditions were as follows: Initial denaturing step, 94°C for 1 minute; 2 cycles of 94°C for 10 seconds and 60°C for 9 minutes; 27 cycles of 94°C for 10 seconds and 62°C for 9 minutes; 62°C for 10 minutes.

The 3' end of the construct, containing a 6.4 Kb fragment that spanned the 3' end of intron 6 to the 5' end of exon 9, was amplified with primer pair HPRT LongArm, forward 5'-CTAGCTAGCGGATTTTGAGCCCCCTTACA-3' and HPRT LongArm, reverse 5'-CTAGCTAGCTGGCCACAGAACTAGAACATTGA-3'. PFU Ultra Polymerase (Stratagene, LaJolla, CA) was used for this PCR. PCR conditions were as follows: Initial denaturing step, 95°C for 2 minutes; 29 cycles of 95°C for 30 seconds, 60°C for 30 seconds and 72°C for 14 minutes; 72°C for 10 minutes.

A SV40 nuclear localizing DNA sequence was PCR amplified from pIRES2-EGFP (BD Biosciences Clontech, Mountain View, CA) with primers SV40, forward 5'GTCGACCCAGCTGTGGAATGTGTGTC-3' and SV40, reverse 5'-GTCGACAACTGGGCGGAGTTAGGG-3' and PFU Ultra Polymerase (Stratagene, LaJolla, CA). The SV40 fragment was subcloned into pPCR-Script AMP SK(+) cloning vector (Stratagene, LaJolla, CA). The pPCR-Script vector containing the SV40 fragment was digested with Sal 1 and the SV40 fragment was gel isolated and cloned into the SAL 1 site of the targeting vector as described above.

The targeting vector was linearized with Not1 enzyme overnight, heat inactivated at 65° for 20 minutes, isopropanol precipitated, washed in 70% ethanol, dried at room temperature for 5 minutes and resuspended in water at a concentration of 1–2 μg/ul.

### Previous Targeting Vectors

All previous targeting vectors, ie, the ones that failed, had the same regions of homology with the exception that pHPRT PURO SV40 had a larger deletion in exon 6. pHPRT +/- used PGK NEO as a selection cassette instead of PGK PURO.

### β-gal Transfection Methods

Lipofectamine (invitrogen, Carlsbad, CA), SuperFect (Qiagen, Hilden, Germany) and FuGENE 6 (Roche, Basel, Switzerland) were all tested for their ability to transfect rhesus fetal fibroblasts with the vector pCMV-sport-β-gal according to the manufacturers' instructions. One group of cells were electroporated at .260 kV, 1 × 10^3 ^μF in 500 μL of basal DMEM with 20 μg of linearized pCMV-sport-B-gal at 4 °C.

After transfection, cells were incubated at 37°C for 7 days and then stained with X-gal. The percent of cells transfected was calculated by comparing the number of blue cells to the number of cells plated.

### Cell Cycle Analysis

hTERT transfected fibroblasts were grown to 70% confluency on 10 cm tissue culture dishes. Five tissue culture dishes containing hTERT transfected fibroblasts received thymidine (Sigma, St. Louis, Mo.), added to a concentration of 2 mM, in the growth media. Another 5 tissue culture dishes received growth media without thymidine.

At time intervals of 12, 18, 24, 30 and 36 hours after treatment, one thymidine-treated dish and one control dish were trypsinized and the cells counted. Pelleted cells were resuspended at one million per ml in Vindelov's Reagent and incubated one hour prior to DNA cell cycle analysis.

### Targeting of HPRT in fibroblasts using electroporation

hTERT transfected and wild type rhesus macaque fibroblasts were grown in Dulbecco's Modified Eagle Media (D-MEM) media (Gibco, Carlsbad, CA) containing 12% fetal bovine serum (FBS) at 37°C, 5% CO_2_. When cells reached 80% confluency on a 300 cm^2 ^flask (Becton Dickinson, Franklin Lakes, NJ), growth media containing 2 mM thymidine (Sigma, St. Louis, MO) was placed on the cells for 24 hours. Cells were then trypsinized and 10 million cells electroporated (Gene Pulser II – Bio-Rad, Philadelphia, PA) with a 20 μg Not1 linearized pHPRT PURO SV40 construct at 4°C (in 800 μl basal D-MEM) in a 4 mm gap cuvette (Bio-Rad, Philadelphia, PA). Cells were pulsed four times at 0.450 kV, 50 μF. Growth media was added and cells plated onto 96 well plates (Becton Dickinson, Franklin Lakes, NJ) at 5000 cells/well.

### Selection Procedures

Four days after electroporation, cells were incubated in selection media containing 7.5 μg/ml puromycin (Sigma, St. Louis, MO). Four days after this, puromycin-resistant (Puro^r^) colonies were counted. These colonies were then placed under selection for HPRT by incubating them in media containing 50 μg/ml 8-Azaguanine (8AG, Sigma, St. Louis, MO) for 6 days. 8AG resistant colonies were passed onto 2 cm^2 ^dishes (Nunc, Rochester, NY). HPRT selection was continued by incubating the 8AG resistant colonies with 80 μM 6-Thioguanine (6TG, Sigma, St. Louis, MO) for 10–14 days.

### PCR screening strategy for HPRT targeted clones

Genomic DNA from 6TG/8AG resistant clones was extracted using the PureGene Genomic DNA Purification Kit (Gentra Systems, Minneapolis, MN). PCR screening was performed with the Advantage GC genomic polymerase (Clontech, Mountain View, CA). The primer pair used was MK HPRT INT5F (5'-TAATGGTGGACTTGTGTTCTAA-3') and MK HPRT EX6R (5'-TTGCGACCTTGACCATCT-3'). Because the reverse primer contained sequence that was deleted from the targeting construct, an amplicon would be expected to be present in wild type cells but not in HPRT targeted cells. The expected size of the PCR product was 3.9 Kb. Ataxia telangiectasia (ATM) primers, AEX1b (5'-GTCAGTCGTGTGGCCGCTCTCTACTGTC-3') AEX6 (5'-CTGCCTGGAGGCTTGTGTTGAGGCTGATAC-3'), were used as positive controls. The ATM PCR product expected size was 5 Kb. The PCR conditions were as follows: Initial denaturing step, 94°C, 1 minute; 2 cycles of 94°C for 10 seconds and 60° for 9 minutes; 27 cycles of 94°C for 10 seconds, 62°C for 9 minutes; 62°C for 10 minutes.

### Cytogenetic analysis

Cytogenetic analysis was performed on the HPRT null fibroblast clones following standard GTW-banding procedures. Specifically, 30 minutes prior to harvest, cells were exposed to Colcemid (0.1 μg/mL). Following hypotonic treatment (0.7% Na citrate for 25 minutes), the preparations were fixed 3 times with methanol:glacial acetic acid (3:1). Metaphase cells were banded with Wright stain and 20 metaphase cells were analyzed. Images were acquired using the Cytovision Image Analysis System (Applied Imaging, Santa Clara, CA) and karyotypes were arranged according to Pearson et al. [43].

## List of abbreviations

6TG: 6-thioguanine; 8AG: 8-azaguanine; ATM: Ataxia telangiectasia mutated gene; B-gal: beta-galactosidase; FBS: fetal bovine serum; G418: Geneticin (neomycin analog); HPRT: hypoxanthine guanine phosphoribosyl transferase

HTERT: human telomerase reverse transcriptase; IRES: internal ribosomal entry site; LB: Luria broth; NEO^r^: neomycin phosphotransferase resistance cassette; NHP: Non human primates;PBS: phosphate buffered saline; PCR: polymerase chain reaction; PGK: phosphoglycerate kinase ; PURO^r^: puromycin resistance cassette; TERT: telomerase reverse transcriptase; UTR: untranslated region

## Authors' contributions

RBN conceived the project. MAZ constructed the vectors and did the PCR screenings. DTM was responsible for cell culture and gene targeting. MM performed comparisons between different chemical transfection methods. MO participated in the TERT experimental design. WS and MS performed the karyotype analysis. DW and SM established primary culture of fibroblasts. All authors read and approved the final manuscript.
